# Unpacking neuropsychiatry and behavioural neurology training: scoping review of core syllabus components

**DOI:** 10.1192/bjb.2025.10184

**Published:** 2026-08

**Authors:** Keishema Kerr, Lauren Burns, Sheldon Benjamin, Eileen M. Joyce, Jasvinder Singh, Jesús Ramírez-Bermúdez, Biba Stanton, Vaughan Bell

**Affiliations:** 1 Clinical, Educational and Health Psychology, University College London, London, UK; 2 Department of Neurology, UMass Chan Medical School, Worcester, Massachusetts, USA; 3 Department of Psychiatry and Behavioral Sciences, UMass Chan Medical School, Worcester, Massachusetts, USA; 4 UCL Queen Square Institute of Neurology, University College London, London, UK; 5 University of Queensland, Brisbane, Australia; 6 Omega Mind Clinics, Brisbane, Australia; 7 Neuropsychiatry Unit, National Institute of Neurology and Neurosurgery, Mexico City, Mexico; 8 Institute of Psychiatry, Psychology and Neuroscience, King’s College London, London, UK; 9 King’s College Hospital NHS Foundation Trust, London, UK; 10 Department of Neuropsychiatry, South London and Maudsley NHS Foundation Trust, London, UK

**Keywords:** Neuropsychiatry, clinical neurology, neuropsychology, education and training, systematic review

## Abstract

**Aims and method:**

There is no consensus on core curriculum content for neuropsychiatry and behavioural neurology training and the breadth of topic coverage is poorly understood. Using a scoping review, we identified 23 unique syllabuses from Australia, Argentina, Chile, Mexico, New Zealand, South Africa, the USA and the UK, and one explicitly international in scope.

**Results:**

Syllabuses addressed a wide range of neuropsychiatric conditions, encompassing not only overlapping psychiatric and neurological disorders, but also functional, behavioural and cognitive disorders. Training integrated knowledge from neuropsychology, philosophy, ethics and social sciences. Core elements included clinical assessment, intervention skills and case management in social and institutional settings. Neuropsychiatry and behavioural neurology training integrates a broad spectrum of knowledge and skills, is aimed at a range of professionals and is delivered as both specialist training and embedded components within core training.

**Clinical implications:**

The core components of neuropsychiatry curricula identified in this study provide a foundation for institutions to develop or enhance their neuropsychiatry training programs.

Neuropsychiatry and behavioural neurology are often considered sister disciplines and involve the treatment, prevention and research of problems at the interface of psychiatry and neurology.^1^ Psychiatric and neurological disorders frequently co-occur and co-present to clinical services. The incidence and prevalence of neurological disorders is markedly raised in people with psychiatric disorders.^2,3^ Conversely, up to half of people referred to neurology clinics meet the diagnostic criteria for a neuropsychiatric diagnosis.^4,5^ Although some co-occurring difficulties may be easily managed by specialists in either discipline, consultation with, or direct management by, clinicians with specialist training in neuropsychiatry is considered best practice or, where best practice is not defined, desirable.^6–8^ We note here that the term ‘neuropsychiatry’ is sometimes used in a wider sense to mean the equivalent of ‘biological psychiatry’ – a field that emphasises neurobiological explanations for psychiatric disorders more widely. Here, however, we are using the term in its more common meaning to refer to the field that deals with conditions that lie at the intersection of psychiatry and neurology.^9,10^

This interface occurs at both the pathological level and the level of clinical practice. Pathologically, neuropsychiatry concerns conditions in which disturbances of brain structure give rise to symptoms traditionally categorised as psychiatric, for example post-stroke mood disorders,^11^ the psychoses of epilepsy^12^ and in autoimmune encephalitis,^13^ or where functional disorders give rise to presentations traditionally categorised as neurological – for example functional neurological disorders.^14^ At the level of clinical practice, neuropsychiatry reflects an integrative approach that draws on neurological, psychiatric and cognitive assessment, often in settings where a single specialty in isolation would not sufficiently capture the complexity of the presentation.^15^

One challenge in recommending specialist training is that there is no consensus about the components of neuropsychiatry and behavioural neurology training, with different authors and organisations citing different requirements for what they consider adequate training.^16^ Some have emphasised an approach that prioritises psychiatric complications of traditionally neurological disorders,^17^ whereas others highlight a wider scope, including areas such as functional neurological disorder, neurodevelopmental disorders and cognitive impairment.^1,18^ Some have argued for research training as core to education in the discipline,^19,20^ whereas others have focused largely on clinical competencies.^21^

There have been a variety of proposals for the place of neuropsychiatry training within medical training as a whole. Suggestions have ranged from encouraging specialists in neuropsychiatry to fully qualify as both psychiatrists and neurologists,^7^ formalising the field as a subspecialty^22^ and integrating the content as a core competency in residency training,^23,24^ to a complete ‘vertical integration’ at all stages of medical training.^25^

Understanding shared and common components of neuropsychiatry and behavioural neurology training programmes would outline a core curriculum as it is currently structured and taught. However, an important obstacle to doing this is that although some curriculum components are published in the peer-reviewed literature, others exist solely as grey literature – either online as part of public-facing course documentation or, in some cases, solely as internal institutional documents.

One way of addressing these obstacles is to conduct a scoping review, which uses a systematic literature search and grey literature searches to map existing literature on the topic.^26^ Consequently, we used a scoping review to understand the common components of neuropsychiatry and behavioural neurology syllabuses across the world to better understand what defines neuropsychiatry as taught to trainees. We deliberately selected an inclusive approach that included academic and clinical neuropsychiatry syllabuses, the latter regardless of target profession, with the aim of clearly distinguishing these characteristics in the analysis.

## Method

This scoping review was conducted in line with the Preferred Reporting Items for Systematic Reviews and Meta-Analyses extension for Scoping Reviews (PRISMA-ScR) guidelines.^27^

### Protocol and registration

The scoping review protocol was pre-registered with INPLASY in September 2023 (registration number INPLASY202390090): https://inplasy.com/inplasy-2023-9-0090/).

As a review of published literature, the study did not require ethical approval.

### Eligibility criteria

We included any paper, study or document giving syllabus contents for a neuropsychiatry and/or behavioural neurology training course. No eligibility limits were imposed for date of the document or language.

### Information sources

Three research and clinical literature databases (PubMed, Embase and CINAHL) were searched on 1 September 2023. The electronic database search was supplemented by reviewing reference lists. Given that many syllabus outlines may not exist in the peer-reviewed literature, we also supplemented the literature database search by searching the web, and we published a request via the Global Neuropsychiatry Group to identify grey literature with syllabus contents. We requested anyone with knowledge of neuropsychiatry or behavioural neurology training courses to inform us. Course leads were then contacted to request syllabus descriptions and courses were identified online to download syllabus descriptions.

### Search

For literature databases, we used the search terms ‘(neuropsychiatr* OR neuro-psychiatry OR neuro-psychiatric OR behavioural neurology OR behavioral neurology) AND (syllabus OR curriculum OR training)’, suitably adapted for each database. We additionally searched the web using similar search terms. The request for syllabuses from the Global Neuropsychiatry Group discussion group was sent to all group members.

### Selection of sources of evidence

References from the search of literature databases were uploaded to Rayyan – an online platform that allows reviewers to collaborate and organise papers to conduct systematic-style reviews. Duplicate records were identified and removed using Rayyan’s inbuilt function. Titles and abstracts were independently screened by authors K.K. and V.B. and disagreements resolved through consensus. Syllabus documents identified through the grey literature identification process were entered directly into the full-text screening stage. The full text of papers and documents was independently assessed by K.K. and V.B. and the final list of documents was identified.

### Data charting and synthesis

Syllabus components were extracted in an iterative process where individual syllabus items were extracted document by document on a coding spreadsheet. If a syllabus item was encountered that was common to a previous code, it was counted under that code. If it was a new item, it was added as a new code and previous documents were checked to ensure that it had not been missed previously. Initial coding was completed by author K.K. and checked by V.B. Higher-level syllabus component categories were coded using the upward coding approach, which involves grouping individual components into semantic categories and transforming the data from a lower to a higher level of abstraction.^28^ Syllabus components were included in higher-level categories non-exclusively, so for example the syllabus component ‘Stroke (and the neuropsychiatric sequelae of)’ contributed to both the ‘Stroke’ and ‘Secondary psychiatric syndromes’ categories. We subsequently calculated frequency tables to identify how frequently syllabus component categories appeared across syllabus documents.

## Results

**Fig. 1:**
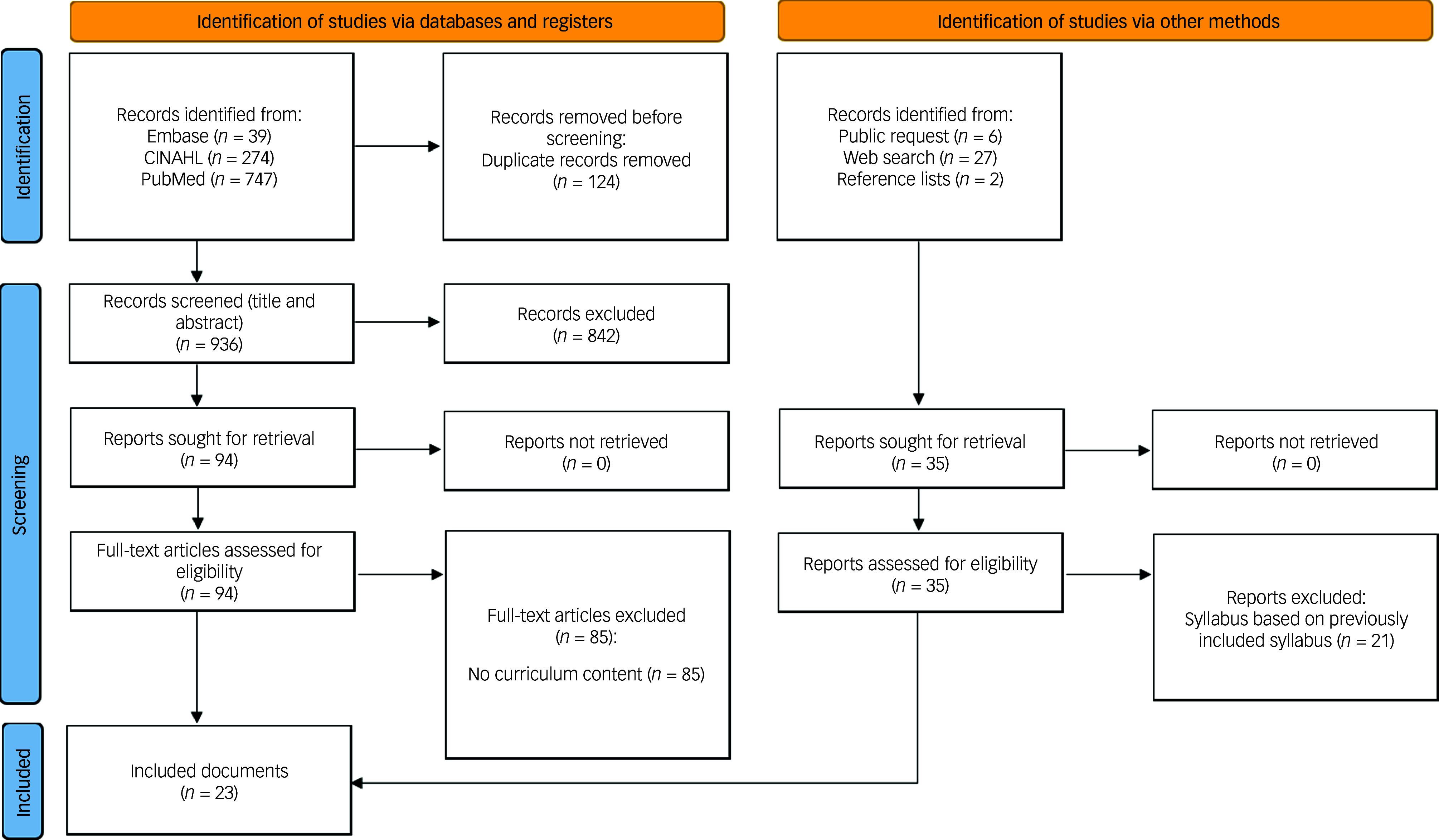
Fig. 1 long description. Preferred Reporting Items for Systematic Reviews and Meta-Analyses (PRISMA) diagram for the scoping review process.

We identified 23 neuropsychiatry and behavioural neurology syllabus documents. The PRISMA diagram for document identification process in the study is illustrated in Fig. 1. Details of all syllabus documents included in the final analysis are listed in in Table 1. We identified syllabus documents from the USA (*k* = 8), UK (*k* = 7), Australia (*k* = 2), Australia and New Zealand (*k* = 1), Argentina (*k* = 1), Chile (*k* = 1), Mexico (*k* = 1), South Africa (*k* = 1) and an international organisation (*k* = 1).

**Table 1:** Syllabus documents included in the scoping review Table 1 long description.

Syllabus name	Year	Country	Source	Type
*Bulletin of US Army Medical Department*: ‘Special Training in Neuropsychiatry’	1946	USA	PRL	Position paper
Benjamin (2004):^29^ ‘Educational issues in neuropsychiatry’	2004	USA	PRL	Position paper
Arciniegas & Kaufer (2006):^1^ ‘Core curriculum for training in behavioral neurology & neuropsychiatry’	2006	USA	PRL	Position paper
Lacy & Hughes (2006):^30^ ‘A neural systems-based neurobiology and neuropsychiatry course: integrating biology, psychodynamics, and psychology in the psychiatric curriculum’	2006	USA	PRL	Position paper
Vaishnavi et al (2009):^31^ ‘Behavioral neurology and neuropsychiatry fellowship training: the Johns Hopkins model’	2009	USA	PRL	Position paper
Sachdev & the Curriculum Committee of the International Neuropsychiatric Association (2010): ‘Core Curriculum in Neuropsychiatry of the International Neuropsychiatric Association’	2010	INT	Book	Position paper
Benjamin (2013):^32^ ‘Educating psychiatry residents in neuropsychiatry and neuroscience’	2013	USA	PRL	Position paper
Joint Royal Colleges of Physicians Training Board: neuropsychiatry section of Curriculum for Neurology Training	2013	UK	Web	Formal, superseded
Benjamin et al (2014):^23^ ‘Neuropsychiatry and neuroscience milestones for general psychiatry trainees’	2014	USA	PRL	Position paper
Sachdev & Mohan (2017):^20^ ‘An international curriculum for neuropsychiatry and behavioural neurology’	2017	AUS	PRL	Position paper
United Council for Neurologic Subspecialties: Behavioral Neurology and Neuropsychiatry Curriculum	2020	USA	PC	Formal, active
Sachdev & Mohan (2020):^33^ ‘Neuropsychiatry curriculum and key clinical competencies’	2020	AUS	Book	Position paper
British Psychological Society: Qualification in Clinical Neuropsychology Handbook and Competency Framework	2022	UK	Web	Formal, active
Joint Royal Colleges of Physicians Training Board: Curriculum for Neurology Training	2022	UK	Web	Formal, active
Royal Australian and New Zealand College of Psychiatrists: Advanced Training in Neuropsychiatry Curriculum	2023	AUS/NZ	PC	Formal, proposed
College of Psychiatrists of South Africa: Sub-specialty Certificate in Neuropsychiatry Syllabus	2023	ZAF	PC	Formal, active
National Institute of Neurology and Neurosurgery: postgraduate course of Advanced Specialty in Neuropsychiatry	2023	MEX	PC	Formal, active
Argentine Neuropsychiatric Association: Advanced Fellowship in Neuropsychiatry and Cognitive Neurology Syllabus	2024	ARG	Web	Formal, active
University of Birmingham: MSc in Clinical Neuropsychiatry	2024	UK	Web	Formal, active
King’s College London: MSc in Clinical Neuropsychiatry Syllabus	2024	UK	PC	Formal, active
University of Chile: Diploma in Adult Clinical Neuropsychiatry	2024	CHL	Web	Formal, active
Association of British Neurologists: Advanced Clinical Fellowship in Behavioural Neurology and Neuropsychiatry	2024	UK	PC	Formal, active
Royal College of Psychiatrists: Syllabus for Higher Training in Neuropsychiatry	2024	UK	Web	Informal, active

ARG, Argentina; AUS, Australia; CHL, Chile; INT, International; MEX, Mexico; NZL, New Zealand; ZAF, South Africa; PRL, peer-reviewed literature; PC, personal communication.

### Syllabus components

Frequency tables for syllabus components are shown in Tables 2, 3, 4 and 5, highlighting topic groupings for neuropsychiatric disorders, fundamental issues, assessment and intervention. Full details of syllabus categories and their constituent syllabus components are shown in Tables S1 to S4 of the supplementary material, available at https://doi.org/10.1192/bjb.2025.10184.

**Table 2:** Frequency of neuropsychiatric disorders in included syllabuses Table 2 long description.

Syllabus component	Syllabus count
Dementia	22
Neurodevelopmental disorders	22
Movement disorders	21
Secondary psychiatric syndromes	21
Mood disorders	21
Neurobehavioural syndromes and challenging behaviour	21
Sleep disorders	20
Epilepsy and seizures	19
Cognitive impairment, selective and general	19
Acquired brain injury	18
Cerebrovascular disorders	18
Functional neurological disorders	17
Psychotic spectrum disorders	17
Demyelinating disorders	16
Neuroinfections	16
Anxiety and stress disorders	16
Substance use and addiction	15
Impulse control disorders	14
Acute and chronic pain disorders	10
Diagnosis of delirium	10
Neuro-oncological disorders	9
Disorders of arousal (e.g. coma, vegetative states)	9
Headache and migraine	9
Malingering and factitious disorders	8
Toxic exposures/ingestions	6
Hydrocephalus	5
Metabolic disorders	5
Neuroendocrine disorders	5
Sexual disorders	4
Motor neuron disease	4
Diagnosis of occupational exposure-related syndromes	2
Chronic fatigue	2
Congenital disorders	1
Nutritional disorders and nutrition in neuropsychiatry	1
Cerebrospinal fluid disorders	1

**Table 3:** Frequency of fundamental issue topics in syllabuses Table 3 long description.

Syllabus component	Syllabus count
Neuroanatomy	21
Neuropsychology and cognition	21
Neurobiology	19
Psychiatry	17
Knowledge of wider specialties	14
Research methods	12
Philosophy and ethics of neuropsychiatry	12
Professional practice issues	12
Genetics	11
Social issues and context	10
Neurology	7
Neurosurgery	2
Psychodynamics	1

**Table 4:** Frequency of assessment topics in syllabuses Table 4 long description.

Syllabus component	Syllabus count
Neuroimaging	23
Diagnosis	23
Neuropsychiatric assessment	22
Cognitive assessment	21
Neurological examination	21
Electroencephalography and electrophysiology	19
Laboratory tests	15
Forensic and risk assessment	9
Visual system assessment	8

**Table 5:** Frequency of intervention topics in syllabuses Table 5 long description.

Syllabus component	Syllabus count
Pharmacological treatments	20
Case management and formulation	20
Psychological interventions	16
Somatic therapies	16
Rehabilitation and neurorehabilitation	14
Social and community interventions	14
Policies, practice and frameworks	11
Acute, crisis and intensive care	2
Immunotherapy	1

### Syllabus formats

Documents included position papers from the peer-reviewed literature (*k* = 8), syllabuses downloaded from websites (*k* = 7), syllabuses received through personal communication (*k* = 6) and position papers published as academic books chapters (*k* = 2). Documents described active training courses (*k* = 11), a formal syllabus for an active training course that had been superseded by a subsequent syllabus (*k* = 1) and a formal syllabus for proposed training courses that had not yet been implemented (*k* = 1). Of these formal training courses, *k* = 7 were open only to physicians, *k* = 1 only to clinical psychologists, *k* = 2 only to neurologists, *k* = 3 only to psychiatrists, and *k* = 4 were academic courses that were open to graduates, including those without clinical training. Within all courses, *k* = 8 involved supervised clinical practice: clinical components of these clinical training courses are outlined in Table 6.

**Table 6:** Clinical training components of neuropsychiatry and behavioural neurology training courses Table 6 long description.

Syllabus name	Clinical training details
Royal Australian and New Zealand College of Psychiatrists: Advanced Training in Neuropsychiatry Curriculum	2-year fellowship programme in a largely or exclusively neuropsychiatric service, or previous 3-year training programme in a centre that offers training in both neurology and psychiatry
National Institute of Neurology and Neurosurgery (Mexico): postgraduate course of Advanced Specialty in Neuropsychiatry	1-year fellowship programme with three 4-month rotations in (a) out-patient neuropsychiatry, (b) liaison, covering neurology, neurosurgery, emergency department and intensive care, and (c) in-patient psychiatry, including patients with primary psychiatric disorders and psychiatric disorders due to neurological disease
United Council for Neurologic Subspecialties: Behavioral Neurology and Neuropsychiatry (USA)	12 months supervised care of patients with focal neurobehavioural syndromes, major neuropsychiatric syndromes and/or cognitive, emotional and behavioural manifestations of neurological conditions. Service placement at the discretion of the programme director and may include out-patient, in-patient, emergency department, other clinic types
College of Psychiatrists of South Africa: Sub-specialty Certificate in Neuropsychiatry	At least 18 months’ full-time experience within an accredited neuropsychiatry unit under the direction of a registered neuropsychiatrist
Royal College of Psychiatrists (UK): Syllabus for Higher Training in Neuropsychiatry	No formal training pathway, but 1-year training posts offered as part of integrated residency training or stand-alone training post
Federation of the Royal Colleges of Physicians of the UK, Joint Royal Colleges of Physicians Training Board: Neurology Training Curriculum (2013, 2022)	No specific neuropsychiatry placement required, but requires neuropsychiatry competencies to be completed as part of neurology residency
Association of British Neurologists: Advanced Clinical Fellowship in Behavioural Neurology and Neuropsychiatry	12 months’ supervised clinical practice, involving brain injury, in-patient neuropsychiatry, general liaison neuropsychiatry and old age liaison neuropsychiatry; in addition, attendance at weekly multidisciplinary team meetings, including neuropsychiatry-focused radiology and electroencephalography, brain injury and less frequently complex somatic symptoms and post-COVID syndrome
British Psychological Society: Qualification in Clinical Neuropsychology	24 months’ clinical practice in neuropsychology, achieving a range of neuropsychology competencies that include neuropsychiatry competencies. Not service specific

## Discussion

In this scoping review of both peer-reviewed and grey literature, we identified 23 unique syllabuses for neuropsychiatry and behavioural neurology courses to identify common course components. The identified course components indicate a thorough coverage of major neuropsychiatric conditions, with the most prevalent disorders covered by most courses and lower prevalence disorders covered less commonly. The covered disorders do not solely focus on the co-presentation of traditionally psychiatric and neurological disorders, but also include functional, behavioural and cognitively defined disorders. Neuropsychiatry and behavioural neurology training was conceptualised as teaching across a range of fundamental issues in mind and brain medicine, neuropsychology, differing degrees of philosophy, ethics and social science. Assessment and intervention components focused on a range of clinical skills and knowledge necessary to work across both mental health and neurological services, with a focus on management of cases in the social and institutional context of the trainee.

We note a clear evolution from proposals in the earlier peer-reviewed literature concerning the necessary components of training to the later development of formal training courses – likely indicating a healthy progression from debate to implementation. Of the syllabuses from formal training programmes, we also note that neuropsychiatry and behavioural neurology training is quite diverse in terms of how it is positioned with regard to clinical training pathways and academic learning. Some training is profession specific – exclusively aimed at physicians, specific medical specialties or clinical psychologists – some is embedded as a component within wider core clinical training, some implements training for a clinical specialisation, and some is designed as academic study, aimed at developing a domain knowledge and is inclusive of wider health professionals and even those with a purely academic interest.

We also note what may at first seem like an anomaly in the frequency of syllabus components. In the ‘fundamental issues’ section the specifically labelled ‘psychiatry’ component appears more frequently than the ‘neurology’ component. However, this appears to be down to naming conventions rather than content per se. There were more syllabuses that classified themselves as ‘neuropsychiatry’ rather than ‘neuropsychiatry and behavioural neurology’ or solely ‘behavioural neurology’, and so some topics were preferentially labelled as ‘psychiatry of’ or ‘neuropsychiatry of’, whereas core neurology components tended not to be named ‘neurology of’. In fact, the analysis of syllabus components as a whole shows that content from psychiatry, neurology, neuropsychology and social science is widely represented. It is also worth noting a broader reality, that in the majority of countries, formal training routes in neuropsychiatry and behavioural neurology are not available, although trainees report varying levels of integration into core training, but express a clear demand for further exposure.^19,34^

Combining internationally sourced syllabus components risks implying that neuropsychiatry and behavioural neurology should be defined in terms of a ‘global average’. Although some common components are likely to be universal, the reported profile should not be seen as prescriptive. The extent to which neuropsychiatry and behavioural neurology need to be adapted to local or regional contexts has been debated with regard to the needs of Latin America^10^ and India and South Asia.^35^ For example, the neuropsychiatry of neglected tropical diseases^36^ is a common focus of clinical and research concern and high priority in some regions,^37^ but less so in others. Therefore, any standardised framework should remain flexible, allowing for core components that transfer across countries while including cultural, clinical and educational adaptations that reflect the priorities of different regions and the needs of different professions within those regions.

It is also useful to compare the state of neuropsychiatry training in light of existing consensus syllabuses for medical specialties. Researchers and professional bodies have published a number of recommendations and consensus guidelines for syllabus contents. These variously range from broad clinical areas, such as clinical experience in neurology,^38^ to specific context-bound interventions, such as pain management in emergency medicine^39^ and ultrasound assessment in critical care.^40^ In parallel, many clinical specialties do not have consensus recommendations and attempts to create them have faltered owing to concerns about adequate applicability across countries and contexts.^41^ Neuropsychiatry may face unique training challenges as it is an interdisciplinary specialty, primarily drawing on approaches from established specialties of psychiatry, neurology and neuropsychology,^15^ and there is a lack of cross-training between neurology and psychiatry since the two fields diverged in the 20th century.^42^ Furthermore, the rapid evolution of neuroscientific understanding and diagnostic technologies requires training programmes to balance foundational knowledge with emerging methodologies, creating additional complexity in curriculum design that may not be present in more established, single-discipline specialties. We suggest that attempts to create a single ‘super curriculum’ may not be successful in neuropsychiatry owing to variation in needs across countries and regions, but awareness of international developments and communication between training curriculum developers is likely to be key in disseminating best practices relevant to local contexts.

### Limitations

We highlight some potential limitations of this scoping review. Although we did not limit syllabuses by language, database searches were completed in English. The databases we selected have a range of non-English language journals abstracted in English, although it is likely that non-English language journals are less likely to be indexed in these databases at all. Although we complemented our database searches by including a request to the Global Neuropsychiatry Group discussion network which, at the time of the query, included about 300 people from across the world with self-described interest in neuropsychiatry, this is primarily an English language group and relies on motivated individuals to respond, and therefore selection bias may have occurred. We identified only three non-English language syllabuses, all from Latin America, but it is unclear whether this reflects a greater presence of neuropsychiatry and behavioural neurology training in the region or simply greater visibility to our search. We also note that there is no standard format for the communication of syllabuses and the documents retrieved varied in their format – from formal descriptions designed to satisfy the accreditation criteria of academic or regulatory bodies to online descriptions aimed at prospective trainees. This may have under-recognised smaller topics, as these were less likely to be included in documents aimed at the public, which appeared more focused on ‘headline’ topics.

We also note that training and syllabus development is an ongoing process and we invite authors of neuropsychiatry and behavioural neurology syllabuses not included in this review, or of those released subsequent to this review, to contact us to help understand how training develops into the future.

## Supporting information

Kerr et al. supplementary material Kerr et al. supplementary material

## Data Availability

Data availability is not applicable to this article as no new data were created or analysed in this study.

## About the authors

**Keishema Kerr** is a clinical psychologist at University College London, London, UK. **Lauren Burns** is a clinical psychologist at University College London, London, UK. **Sheldon Benjamin** is a Professor of Psychiatry and Neurology at the Department of Neurology and Department of Psychiatry and Behavioral Sciences, UMass Chan Medical School, Worcester, Massachusetts, USA. **Eileen M. Joyce** is a Professor of Neuropsychiatry at UCL Queen Square Institute of Neurology, University College London, London, UK. **Jasvinder Singh** is a neuropsychiatrist at the University of Queensland, Brisbane, and Omega Mind Clinics, Brisbane, Australia. **Jesús Ramírez-Bermúdez** is a clinician, researcher, and professor at the Neuropsychiatry Unit, National Institute of Neurology and Neurosurgery, Mexico City, Mexico. **Biba Stanton** is a consultant neurologist and visiting senior lecturer at the Institute of Psychiatry, Psychology and Neuroscience, King’s College London, and King’s College Hospital NHS Foundation Trust, London, UK. **Vaughan Bell** is a professor of clinical psychology and cognitive neuropsychiatry at University College London, London, UK.

## Supplementary material

The supplementary material is available online at https://doi.org/10.1192/bjb.2025.10184.

## Fig. 1 Long description

A flowchart illustrating the process of identifying and screening studies for a scoping review. The flowchart is divided into two main sections: Identification of studies via databases and registers, and Identification of studies via other methods. The identification section shows records identified from Embase, CINAHL, and PubMed, with duplicate records removed. The screening section details the process of screening titles and abstracts, seeking reports for retrieval, and assessing full-text articles for eligibility. The flowchart also includes decision points where records are excluded based on specific criteria. The final outcome is the inclusion of documents that meet the eligibility criteria.

Navigate back to Fig. 1.

## Table 1 Long description

A table listing neuropsychiatry and behavioral neurology syllabus documents by year, country, source, and type. The table has 23 rows and 5 columns. Column headers are Syllabus name, Year, Country, Source, and Type. Row 1: Bulletin of US Army Medical Department: 'Special Training in Neuropsychiatry', 1946, USA, PRL, Position paper. Row 2: Benjamin (2004): 'Educational issues in neuropsychiatry', 2004, USA, PRL, Position paper. Row 3: Arciniegas & Kaufer (2006): 'Core curriculum for training in behavioral neurology & neuropsychiatry', 2006, USA, PRL, Position paper. Row 4: Lacey & Hughes (2006): 'A neural systems-based neurobiology and neuropsychiatry course: integrating biology, psychodynamics, and psychology in the psychiatric curriculum', 2006, USA, PRL, Position paper. Row 5: Vaishnavi et al (2009): 'Behavioral neurology and neuropsychiatry fellowship training: the Johns Hopkins model', 2009, USA, PRL, Position paper. Row 6: Sachdev & the Curriculum Committee of the International Neuropsychiatric Association (2010): 'Core Curriculum in Neuropsychiatry of the International Neuropsychiatric Association', 2010, INT, Book, Position paper. Row 7: Benjamin (2013): 'Educating psychiatry residents in neuropsychiatry and neuroscience', 2013, USA, PRL, Position paper. Row 8: Joint Royal Colleges of Physicians Training Board: neuropsychiatry section of Curriculum for Neurology Training, 2013, UK, Web, Formal, superseded. Row 9: Benjamin et al (2014): 'Neuropsychiatry and neuroscience milestones for general psychiatry trainees', 2014, USA, PRL, Position paper. Row 10: Sachdev & Mohan (2017): 'An international curriculum for neuropsychiatry and behavioural neurology', 2017, AUS, PRL, Position paper. Row 11: United Council for Neurologic Subspecialties: Behavioral Neurology and Neuropsychiatry Curriculum, 2020, USA, PC, Formal, active. Row 12: Sachdev & Mohan (2020): 'Neuropsychiatry curriculum and key clinical competencies', 2020, AUS, Book, Position paper. Row 13: British Psychological Society: Qualification in Clinical Neuropsychology Handbook and Competency Framework, 2022, UK, Web, Formal, active. Row 14: Joint Royal Colleges of Physicians Training Board: Curriculum for Neurology Training, 2022, UK, Web, Formal, active. Row 15: Royal Australian and New Zealand College of Psychiatrists: Advanced Training in Neuropsychiatry Curriculum, 2023, AUS/NZ, PC, Formal, proposed. Row 16: College of Psychiatrists of South Africa: Sub-specialty Certificate in Neuropsychiatry Syllabus, 2023, ZAF, PC, Formal, active. Row 17: National Institute of Neurology and Neurosurgery: postgraduate course of Advanced Specialty in Neuropsychiatry, 2023, MEX, PC, Formal, active. Row 18: Argentine Neuropsychiatric Association: Advanced Fellowship in Neuropsychiatry and Cognitive Neurology Syllabus, 2024, ARG, Web, Formal, active. Row 19: University of Birmingham: MSc in Clinical Neuropsychiatry, 2024, UK, Web, Formal, active. Row 20: King's College London: MSc in Clinical Neuropsychiatry Syllabus, 2024, UK, PC, Formal, active. Row 21: University of Chile: Diploma in Adult Clinical Neuropsychiatry, 2024, CHL, Web, Formal, active. Row 22: Association of British Neurologists: Advanced Clinical Fellowship in Behavioural Neurology and Neuropsychiatry, 2024, UK, PC, Formal, active. Row 23: Royal College of Psychiatrists: Syllabus for Higher Training in Neuropsychiatry, 2024, UK, Web, Informal, active.

Navigate back to Table 1.

## Table 2 Long description

A table with two columns and thirty rows. The first column is labeled Syllabus component and the second column is labeled Syllabus count. The table lists various syllabus components along with their respective counts. Row 1: Dementia, 22. Row 2: Neurodevelopmental disorders, 22. Row 3: Movement disorders, 21. Row 4: Secondary psychiatric syndromes, 21. Row 5: Mood disorders, 21. Row 6: Neurobehavioural syndromes and challenging behaviour, 21. Row 7: Sleep disorders, 20. Row 8: Epilepsy and seizures, 19. Row 9: Cognitive impairment, selective and general, 19. Row 10: Acquired brain injury, 18. Row 11: Cerebrovascular disorders, 18. Row 12: Functional neurological disorders, 17. Row 13: Psychotic spectrum disorders, 17. Row 14: Demyelinating disorders, 16. Row 15: Neuroinfections, 16. Row 16: Anxiety and stress disorders, 16. Row 17: Substance use and addiction, 15. Row 18: Impulse control disorders, 14. Row 19: Acute and chronic pain disorders, 10. Row 20: Diagnosis of delirium, 10. Row 21: Neuro-oncological disorders, 9. Row 22: Disorders of arousal (e.g. coma, vegetative states), 9. Row 23: Headache and migraine, 9. Row 24: Malingering and factitious disorders, 8. Row 25: Toxic exposures/ingestions, 6. Row 26: Hydrocephalus, 5. Row 27: Metabolic disorders, 5. Row 28: Neuroendocrine disorders, 5. Row 29: Sexual disorders, 4. Row 30: Motor neuron disease, 4. Row 31: Diagnosis of occupational exposure-related syndromes, 2. Row 32: Chronic fatigue, 2. Row 33: Congenital disorders, 1. Row 34: Nutritional disorders and nutrition in neuropsychiatry, 1. Row 35: Cerebrospinal fluid disorders, 1.

Navigate back to Table 2.

## Table 3 Long description

A table comparing the frequency of fundamental issue topics in syllabuses across different components. The table has two columns: Syllabus component and Syllabus count. It contains 13 rows. Row 1: Neuroanatomy, 21. Row 2: Neuropsychology and cognition, 21. Row 3: Neurobiology, 19. Row 4: Psychiatry, 17. Row 5: Knowledge of wider specialties, 14. Row 6: Research methods, 12. Row 7: Philosophy and ethics of neuropsychiatry, 12. Row 8: Professional practice issues, 12. Row 9: Genetics, 11. Row 10: Social issues and context, 10. Row 11: Neurology, 7. Row 12: Neurosurgery, 2. Row 13: Psychodynamics, 1.

Navigate back to Table 3.

## Table 4 Long description

A table comparing the frequency of various assessment topics in syllabuses. The table has two columns: Syllabus component and Syllabus count. It contains nine rows with the following data: Row 1: Neuroimaging, 23; Row 2: Diagnosis, 23; Row 3: Neuropsychiatric assessment, 22; Row 4: Cognitive assessment, 21; Row 5: Neurological examination, 21; Row 6: Electroencephalography and electrophysiology, 19; Row 7: Laboratory tests, 15; Row 8: Forensic and risk assessment, 9; Row 9: Visual system assessment, 8.

Navigate back to Table 4.

## Table 5 Long description

A table comparing the frequency of various syllabus components. The table has two columns: Syllabus component and Syllabus count. It contains nine rows with the following data: Row 1: Syllabus component, Pharmacological treatments; Syllabus count, 20. Row 2: Syllabus component, Case management and formulation; Syllabus count, 20. Row 3: Syllabus component, Psychological interventions; Syllabus count, 16. Row 4: Syllabus component, Somatic therapies; Syllabus count, 16. Row 5: Syllabus component, Rehabilitation and neurorehabilitation; Syllabus count, 14. Row 6: Syllabus component, Social and community interventions; Syllabus count, 14. Row 7: Syllabus component, Policies, practice and frameworks; Syllabus count, 11. Row 8: Syllabus component, Acute, crisis and intensive care; Syllabus count, 2. Row 9: Syllabus component, Immunotherapy; Syllabus count, 1.

Navigate back to Table 5.

## Table 6 Long description

A table comparing clinical training details for neuropsychiatry and behavioral neurology across different institutions. The table has 2 columns and 8 rows. Column headers are Syllabus name and Clinical training details. Row 1: Royal Australian and New Zealand College of Psychiatrists: Advanced Training in Neuropsychiatry Curriculum, 2-year fellowship programme in a largely or exclusively neuropsychiatric service, or previous 3-year training programme in a centre that offers training in both neurology and psychiatry. Row 2: National Institute of Neurology and Neurosurgery (Mexico): postgraduate course of Advanced Specialty in Neuropsychiatry, 1-year fellowship programme with three 4-month rotations in (a) out-patient neuropsychiatry, (b) liaison, covering neurology, neurosurgery, emergency department and intensive care, and (c) in-patient psychiatry, including patients with primary psychiatric disorders and psychiatric disorders due to neurological disease. Row 3: United Council for Neurologic Subspecialties: Behavioral Neurology and Neuropsychiatry (USA), 12 months supervised care of patients with focal neurobehavioural syndromes, major neuropsychiatric syndromes and/or cognitive, emotional and behavioural manifestations of neurological conditions. Service placement at the discretion of the programme director and may include out-patient, in-patient, emergency department, other clinic types. Row 4: College of Psychiatrists of South Africa: Sub-specialty Certificate in Neuropsychiatry, At least 18 months’ full-time experience within an accredited neuropsychiatry unit under the direction of a registered neuropsychiatrist. Row 5: Royal College of Psychiatrists (UK): Syllabus for Higher Training in Neuropsychiatry, No formal training pathway, but 1-year training posts offered as part of integrated residency training or stand-alone training post. Row 6: Federation of the Royal Colleges of Physicians of the UK, Joint Royal Colleges of Physicians Training Board: Neurology Training Curriculum (2013, 2022), No specific neuropsychiatry placement required, but requires neuropsychiatry competencies to be completed as part of neurology residency. Row 7: Association of British Neurologists: Advanced Clinical Fellowship in Behavioural Neurology and Neuropsychiatry, 12 months’ supervised clinical practice, involving brain injury, in-patient neuropsychiatry, general liaison neuropsychiatry and old age liaison neuropsychiatry; in addition, attendance at weekly multidisciplinary team meetings, including neuropsychiatry-focused radiology and electroencephalography, brain injury and less frequently complex somatic symptoms and post-COVID syndrome. Row 8: British Psychological Society: Qualification in Clinical Neuropsychology, 24 months’ clinical practice in neuropsychology, achieving a range of neuropsychology competencies that include neuropsychiatry competencies. Not service specific.

Navigate back to Table 6.

## References

[1] Arciniegas DB , Kaufer DI. Core curriculum for training in behavioral neurology & neuropsychiatry. JNP 2006; 18: 6–13. 10.1176/jnp.18.1.6 16525065

[2] Hesdorffer DC. Comorbidity between neurological illness and psychiatric disorders. CNS Spectr 2016; 21: 230–8. 26898322 10.1017/S1092852915000929

[3] Nuyen J , Schellevis FG , Satariano WA , Spreeuwenberg PM , Birkner MD , van den Bos GAM , et al. Comorbidity was associated with neurologic and psychiatric diseases: a general practice-based controlled study. J Clin Epidemiol 2006; 59: 1274–84. 17098570 10.1016/j.jclinepi.2006.01.005

[4] Carson AJ , Ringbauer B , MacKenzie L , Warlow C , Sharpe M. Neurological disease, emotional disorder, and disability: they are related: a study of 300 consecutive new referrals to a neurology outpatient department. J Neurol Neurosurg Psychiatry 2000; 68: 202–6. 10644788 10.1136/jnnp.68.2.202 PMC1736760

[5] Jabbar F , Doherty A , Duffy R , Aziz M , Casey P , Sheehan J , et al. The role of a neuropsychiatry clinic in a tertiary referral teaching hospital: a 2-year study. Irish J Psychol Med 2014; 31: 271–3. 10.1017/ipm.2014.38 30189503

[6] Agrawal N , Fleminger S , Ring H , Deb S. Neuropsychiatry in the UK: planning the service provision for the 21st century. Psychiatr Bull 2008; 32: 303–6.

[7] Benjamin S. Dual residency training in neurology and psychiatry: history and current practice. JNP 2024; 36: 11–21. 10.1176/appi.neuropsych.21110271 37727060

[8] Sachdev P. Whither neuropsychiatry? JNP 2005; 17: 140–1. 10.1176/jnp.17.2.140 15939966

[9] Lishman WA. What is neuropsychiatry? J Neurol Neurosurg Psychiatry 1992; 55: 983–5. 1469416 10.1136/jnnp.55.11.983 PMC1015277

[10] Ramírez-Bermúdez J , Juarez FPG , Aliseda A. Neuropsychiatric constructs as bridges between psychopathology and neuropathology: a medical perspective. Rev Phil Psych, 2025; 16: 909–31.

[11] Ferro JM , Caeiro L , Figueira ML. Neuropsychiatric sequelae of stroke. Nat Rev Neurol 2016; 12: 269–80. 27063107 10.1038/nrneurol.2016.46

[12] Mula M , Kanner AM , Young AH , Giorgio AD , Schulze-Bonhage A , Trinka E. Epilepsy and psychosis: navigating through a complex intersection. BJPsych Open 2025; 11: e127. 40566953 10.1192/bjo.2025.70 PMC12247078

[13] Pollak TA , Coutinho E , Palmer-Cooper E , Vincent A. Inflammatory and autoimmune disorders in neuropsychiatry. In Oxford Textbook of Neuropsychiatry (eds N Agrawal , R Faruqui , M Bodani , N Agrawal , R Faruqui , M Bodani ): 245–58. Oxford University Press, 2020.

[14] Perez DL , Aybek S , Popkirov S , Kozlowska K , Stephen CD , Anderson J , et al. A review and expert opinion on the neuropsychiatric assessment of motor functional neurological disorders. JNP 2021; 33: 14–26. 10.1176/appi.neuropsych.19120357 32778007

[15] Trapp NT , Martyna MR , Siddiqi SH , Bajestan SN. The neuropsychiatric approach to the assessment of patients in neurology. Semin Neurol 2022; 42: 88–106. 35477181 10.1055/s-0042-1745741 PMC9177704

[16] Perez DL. Doubling down on combined neurology-psychiatry residency training and behavioral neurology & neuropsychiatry fellowship training. JNP 2024; 36: 78–9. 10.1176/appi.neuropsych.20230065 38226911

[17] Agrawal N. Neuropsychiatry. BMJ 2004; 328: 0403110.

[18] Bateman JR , Josephy-Hernandez S , Apostolova LG , Benjamin S , Barrett AM , Boeve BF , et al. Promoting growth in behavioral neurology: a path forward. Cogn Behav Neurol 2024; 37: 49. 38717325 10.1097/WNN.0000000000000368

[19] Molina-Ruiz R , Nakagami Y , Mörkl S , Vargas M , Shalbafan M , Chang JPC , et al. Training in neuropsychiatry: views of early career psychiatrists from across the world. BJPsych Bull 2024; 48: 78–84. 37395121 10.1192/bjb.2023.32 PMC10985715

[20] Sachdev P , Mohan A. An international curriculum for neuropsychiatry and behavioural neurology. Rev Colomb Psiquiatr 2017; 46: 18–27. 29037334 10.1016/j.rcp.2017.05.001

[21] Shalev D , Jacoby N. Neurology training for psychiatry residents: practices, challenges, and opportunities. Acad Psychiatry 2019; 43: 89–95. 29777396 10.1007/s40596-018-0932-4

[22] Silver JM. Behavioral neurology and neuropsychiatry is a subspecialty. JNP 2006; 18: 146–8. 10.1176/jnp.2006.18.2.146 16720790

[23] Benjamin S , Widge A , Shaw K. Neuropsychiatry and neuroscience milestones for general psychiatry trainees. Acad Psychiatry 2014; 38: 275–82. 24715675 10.1007/s40596-014-0112-0

[24] Torous J , Stern AP , Padmanabhan JL , Keshavan MS , Perez DL. A proposed solution to integrating cognitive-affective neuroscience and neuropsychiatry in psychiatry residency training: the time is now. Asian J Psychiatry 2015; 17: 116–21. 10.1016/j.ajp.2015.05.007 26054985

[25] Mitchell AJ , Agrawal N. Training in neuropsychiatry: is it time to reintegrate into mainstream psychiatry? Psychiatr Bull 2005; 29: 361–4.

[26] Munn Z , Peters MDJ , Stern C , Tufanaru C , McArthur A , Aromataris E. Systematic review or scoping review? Guidance for authors when choosing between a systematic or scoping review approach. BMC Med Res Methodol 2018; 18: 143. 30453902 10.1186/s12874-018-0611-x PMC6245623

[27] Tricco AC , Lillie E , Zarin W , O’Brien KK , Colquhoun H , Levac D , et al. PRISMA extension for scoping reviews (PRISMA-ScR): checklist and explanation. Ann Intern Med 2018; 169: 467–73. 30178033 10.7326/M18-0850

[28] Jansen H. The logic of qualitative survey research and its position in the field of social research methods. FQS 2010; 11(2): art 11.

[29] Benjamin S. Educational issues in neuropsychiatry. Psychiatr Times 2004; 21: 72.

[30] Lacy T , Hughes JD. A neural systems-based neurobiology and neuropsychiatry course: integrating biology, psychodynamics, and psychology in the psychiatric curriculum. Acad Psychiatry 2006; 30: 410–5. 17021150 10.1176/appi.ap.30.5.410

[31] Vaishnavi S , Rosenblatt A , Rabins P , Lyketsos C , Rao V. Behavioral neurology and neuropsychiatry fellowship training: the Johns Hopkins model. J Neuropsychiatry Clin Neurosci 2009; 21: 335–41. 19776316 10.1176/jnp.2009.21.3.335

[32] Benjamin S. Educating psychiatry residents in neuropsychiatry and neuroscience. Int Rev Psychiatry 2013; 25: 265–75. 23859089 10.3109/09540261.2013.786689

[33] Sachdev PS , Mohan A. Neuropsychiatry curriculum and key clinical competencies. In Oxford Textbook of Neuropsychiatry (eds N Agrawal , R Faruqui , M Bodani ): 141–50. Oxford Academic, 2020.

[34] Costello H , Baum M , Watson C , Badenoch JB , Burchill E , Rogers JP , et al. A national survey of neuropsychiatry training experiences. BJPsych Bull [Epub ahead of print] 4 June 2025. Available from: 10.1192/bjb.2025.34. PMC13150525 40462251

[35] Krishnamoorthy ES , Misra V. Neuropsychiatry service provision in India and South Asia. In Oxford Textbook of Neuropsychiatry (eds N Agrawal , R Faruqui , M Bodani ): 545–50. Oxford University Press, 2020.

[36] Berkowitz AL , Raibagkar P , Pritt BS , Mateen FJ. Neurologic manifestations of the neglected tropical diseases. J Neurol Sci 2015; 349: 20–32. 25623803 10.1016/j.jns.2015.01.001

[37] Rosa da RF , Junior FD de PG , Gondim F de AA . Arquivos de Neuro-Psiquiatria, 80 years: part 1 (1943–1962). Arq Neuro-Psiquiatr 2025; 83: 25–32. 10.1055/s-0045-1804917 PMC11922621 40107258

[38] Safdieh JE , Govindarajan R , Gelb DJ , Odia Y , Soni M. Core curriculum guidelines for a required clinical neurology experience. Neurology 2019; 92: 619–26. 30796141 10.1212/WNL.0000000000007187 PMC6453766

[39] Poon SJ , Nelson LS , Hoppe JA , Perrone J , Sande MK , Yealy DM , et al. Consensus-based recommendations for an emergency medicine pain management curriculum. J Emerg Med 2016; 51: 147–54. 27369855 10.1016/j.jemermed.2016.05.009

[40] Neri L , Storti E , Lichtenstein D. Toward an ultrasound curriculum for critical care medicine. Crit Care Med 2007; 35: S290. 17446790 10.1097/01.CCM.0000260680.16213.26

[41] Weisz G , Nannestad B. The World Health Organization and the global standardization of medical training, a history. Global Health 2021; 17: 96. 34454517 10.1186/s12992-021-00733-0 PMC8397872

[42] Casper ST. The Neurologists: A History of a Medical Specialty in Modern Britain, c. 1789–2000. Manchester University Press, 2016.

